# Bovine Parainfluenza Virus Type 3 Infection Reprograms the Bovine Serum Lipidome Associated with Phosphatidylinositol Depletion and Sphingolipid Axis Activation

**DOI:** 10.3390/microorganisms14010252

**Published:** 2026-01-21

**Authors:** Shubo Wen, Jiongjie Zhang, Na Lu, Deqing Tian, Lingpin Meng, Zheng Gao, Yang Song

**Affiliations:** 1 College of Animal Science and Technology, Inner Mongolia Minzu University, Tongliao 028000, China; shubowen@imun.edu.cn (S.W.); zjj6224@163.com (J.Z.); 14719672694@163.com (N.L.); 18536496055@163.com (D.T.); meng02101689@163.com (L.M.); gzheng4955@gmail.com (Z.G.); 2 Inner Mongolia Autonomous Region Engineering Research Center for Disease Prevention and Control of Beef Cattle, Tongliao 028000, China

**Keywords:** BPIV3, phosphatidylcholine, phosphatidylinositol

## Abstract

Bovine Parainfluenza Virus Type 3 (BPIV3) is a critical pathogen in the Bovine Respiratory Disease Complex (BRDC), leading to significant economic losses in the cattle industry. However, the metabolic reprogramming induced by BPIV3 in cattle remains poorly understood. This study aimed to investigate the impact of BPIV3 infection on the serum metabolome of Simmental cattle using untargeted metabolomics and ultra-performance liquid chromatography-quadrupole time-of-flight mass spectrometry (UPLC-QTOF-MS/MS). The results revealed significant alterations in the lipidome, including the upregulation of phosphatidylcholine (PC) and phosphatidylglycerol (PG), and the downregulation of phosphatidylinositol (PI). Sphingolipid metabolism also showed considerable changes, with increased levels of Trihexosylceramide and D-erythro-Sphingosine C-17. Furthermore, metabolic pathway analysis identified enriched pathways related to lipid metabolism, amino acid metabolism, and energy sensing. These findings suggest that BPIV3 infection induces substantial shifts in lipid metabolism, which may facilitate viral replication and immune evasion. Our results provide a deeper understanding of the metabolic changes in BPIV3-infected cattle and propose potential targets for therapeutic intervention.

## 1. Introduction

Bovine Respiratory Disease Complex (BRDC) poses a critical health threat to the global cattle industry, causing annual economic losses exceeding $1 billion [1]. This complex disease is driven by a combination of viral, bacterial, and environmental factors, with Bovine Parainfluenza Virus 3 (BPIV3) being one of the key viral contributors, detected in 20% of clinical cases [2]. BPIV3 enters respiratory epithelial cells through its hemagglutinin-neuraminidase (HN) protein, which binds to sialic acid receptors on cell surfaces. The virus then exploits cholesterol-rich lipid raft structures for membrane fusion and cellular invasion [3]. BPIV3 infection induces severe immune suppression and respiratory inflammation, which significantly increases the incidence of pneumonia in calves by three-fold and raises mortality rates to as high as 20% [4]. In high-density farming systems, breeds such as Simmental cattle are particularly vulnerable to BPIV3, as transportation stress and mixed-group management practices further facilitate virus transmission.

Despite significant advances in characterizing BPIV3 at the molecular level, the mechanisms by which it hijacks host metabolic networks remain poorly understood. Existing evidence indicates that the virus upregulates autophagy-related gene Beclin1 by activating endoplasmic reticulum stress (ER stress), using autophagosomes to facilitate its replication [5]; Disruption of cholesterol-rich lipid rafts inhibits over 90% of viral invasion, while cholesterol supplementation restores infectivity [3]. This highlights the virus’s reliance on host lipid metabolism. Traditional diagnostic tools, such as nucleic acid testing and serological assays, are limited in detecting only the presence of the pathogen and fail to capture early pathological changes [6]. In contrast, metabolomics offers a powerful approach to profiling dynamic changes in small-molecule metabolites (<1 kDa), providing new insights into pathogen-host interactions [7]. However, specific metabolic changes induced by BPIV3 have yet to be defined.

Simmental cattle hold unique research value due to their distinct metabolic traits. This breed is prone to glycolipid metabolic disorders under transport stress, which may exacerbate BPIV3 pathogenicity [8], Furthermore, the immune-metabolic regulatory networks in Simmental cattle display breed-specific traits [9], underscoring the need for targeted studies. In this study, we aim to integrate ultra-performance liquid chromatography-quadrupole time-of-flight mass spectrometry (UPLC-QTOF-MS/MS) with multivariate statistical analysis to comprehensively explore the impact of BPIV3 infection on the serum metabolome of Simmental cattle.

## 2. Materials and Methods

### 2.1. Sample Collection

The experiment was conducted at a large-scale Simmental beef cattle farm in Kulun, Tongliao City, where 200 approximately 10-month-old Simmental heifers were selected for this study. Each animal was assigned a unique identification number, and relevant health information was recorded. Nasal swabs and blood samples were collected from all animals. The nasal swabs were stored in ice boxes, while the blood samples were allowed to stand at room temperature for 30 min before being transported to the Preventive Veterinary Medicine Laboratory at Inner Mongolia Minzu University. Upon arrival, the blood samples were centrifuged at 3000 rpm for 15 min to separate the serum, which was subsequently stored at −80 °C for further analysis.

### 2.2. PCR Detection for Pathogens in the Nasal Swab Samples

Polymerase chain reaction (PCR) testing was performed on the nasal swab samples of the 200 selected cattle to detect BPIV3, IBRV, BVDV, and BRSV, using primers listed in Table 1. Based on the pathogen infection status of the cattle, 18 heifers were selected for further study. Of these, 9 heifers were classified as healthy, with all pathogen tests being negative (Healthy group, *n* = 9), while the remaining 9 heifers exhibited coughing symptoms and tested positive for BPIV3, with negative results for BVDV, BRSV, and IBRV (Infected group, *n* = 9). The corresponding serum samples from these 18 heifers were then sent to BioMarker Technologies Co. (Beijing Biomarker Technologies Co., Ltd., Beijing, China) for untargeted metabolomics analysis.

### 2.3. Plasma Metabolomics Profiling

The serum samples were initially extracted using the methanol-internal standard method. A 100 μL sample was mixed with 300 μL of methanol and 20 μL of internal standard, followed by sonication for 10 min. The mixture was then incubated at −20 °C for 1 h and centrifuged at 4 °C at high speed. After centrifugation, 200 μL of the supernatant was collected. Some samples were pooled to serve as the 0 °C control, and selected samples were analyzed.

The serum samples were analyzed using an ultra-high-performance liquid chromatography system (Waters Acquity I-Class PLUS UHPLC) coupled with a high-resolution quadrupole time-of-flight mass spectrometer (Xevo G2-XS QToF). Chromatographic separation was carried out on an Acquity UPLC HSS T3 column (1.8 μm, 2.1 × 100 mm), maintained at 40 °C. The mobile phase consisted of (A) water with 0.1% formic acid and (B) acetonitrile. A gradient elution program was employed as follows: initial conditions were set at 98% A, held for 10 min, followed by a linear gradient to 98% B over 5 min, which was then held for 3 min. The system was then returned to the initial conditions and equilibrated for 5 min. The flow rate was set at 0.4 mL/min, and the injection volume was 2 μL.

Mass spectrometric detection was performed in MSe mode with dual-channel data acquisition to facilitate comprehensive metabolite profiling. In positive ion mode, the capillary voltage was set to +2000 V, and in negative ion mode, it was set to −1500 V. The source temperature was maintained at 150 °C, and the desolvation gas temperature was set to 500 °C with a flow rate of 800 L/h. Data acquisition utilized alternating low and high collision energies: low energy was set at 2 V for precursor ion detection, while high energy was ramped from 10 to 40 V to obtain fragment ion information. The scan time was 0.2 s per spectrum, and the mass range was set from 50 to 1200 Da.

Raw data were processed using Progenesis QI 2.0 software, where features from both positive and negative ionization modes were combined, aligned, and de-duplicated to generate a consolidated list of metabolites for subsequent statistical analysis.

### 2.4. Statistical Analysis

The raw data from LC-MS analysis were imported into the metabolomics data processing software Progenesis QI for subsequent analysis. Orthogonal partial least squares discriminant analysis (OPLS-DA) was performed to identify group differences, and a volcano plot was generated. Differential metabolites were determined based on the variable importance in projection (VIP) values and p-values derived from the OPLS-DA model. The selection criteria for differential metabolites were set as VIP > 1 and *p* < 0.05. These differential metabolites were then annotated for metabolic pathways using the Kyoto Encyclopedia of Genes and Genomes (KEGG) database to identify the associated metabolic pathways.

## 3. Results

### 3.1. Pathogen Confirmation and Cohort Definition

**Figure 1:**
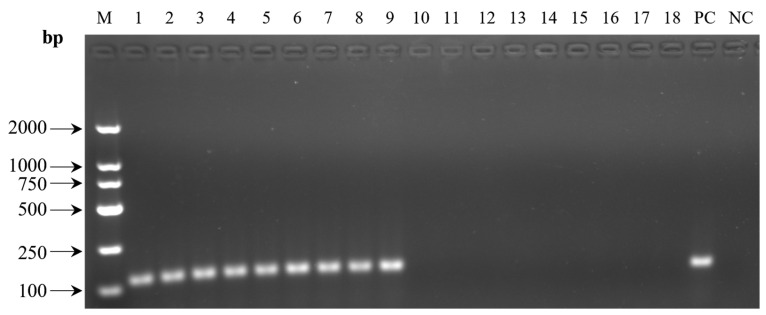
Result of PCR detection for BPIV3. M DL2000 marker; 1–18 the nasal swab samples of Simmental heifers; PC the positive control; NC the negative control.

The results of agarose gel electrophoresis showed that the BPIV3 PCR products from the nasal swab cDNA samples of the infected group (lanes 1–9) displayed clear specific bands at the expected molecular weight (122 bp). In contrast, no bands were observed in the target region for the nasal swab cDNA samples from the healthy group (lanes 10–18) and the negative control (NC, lane 19) (Figure 1).

### 3.2. Global Metabolome Overview and Data Quality

**Figure 2:**
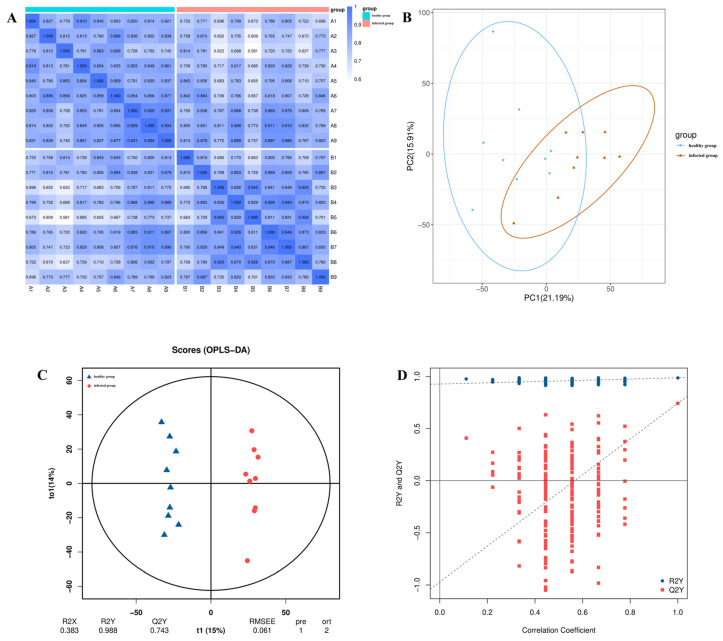
Sample Grouping and Model Validation Based on PCA and OPLS-DA Analysis. (**A**) Correlation heatmap between samples under mixed ion mode. Both axes represent sample names, colored by group. The color intensity in each cell indicates the Pearson correlation coefficient (r). Bold numbers denote correlations that are statistically significant (*p* < 0.05). (**B**) Principal component analysis (PCA) score plot. The X-axis (PC1) and Y-axis (PC2) explain 21.19% and 15.91% of the variance, respectively. Each point represents an individual sample, colored by group. Dashed ellipses outline the 95% confidence interval for each group, showing a clear separation trend. (**C**) Orthogonal partial least squares-discriminant analysis (OPLS-DA) score plot (R2X = 0.383, R2Y = 0.988, Q2Y = 0.743, RMSEP = 0.061), demonstrating clear inter-group discrimination. The dashed ellipse represents the 95% confidence interval for the modeled groups. (**D**) Permutation test plot (n = 200) for validating the OPLS-DA model. The horizontal dashed line indicates the threshold for a significant model (Q2 > 0.5). The results confirm the robustness of the model, as most permuted Q2 values (red squares) are below the original R2Y (blue square).

Through untargeted metabolomics analysis, we conducted a comprehensive examination of the serum metabolite profiles of BPIV3-infected and healthy Simmental cattle. To identify differences in metabolite accumulation due to BPIV3 infection, correlation analysis (Figure 2A) and principal component analysis (PCA) (Figure 2B) were performed on the samples. In the correlation analysis, an r value closer to 1 indicates a stronger correlation between duplicate samples. The data reveal that the similarity within the sample group is relatively high, whereas the similarity between groups is lower compared to the intra-group variance. The PCA results show that in the mixed ion mode, PC1 explained 21.19% of the variance, while PC2 explained 15.91%, with the two principal components together accounting for 37.1% of the total variance. Although the cumulative explained variance is moderate, further orthogonal partial least squares discriminant analysis (OPLS-DA) (Figure 2C) and the permutation test (Figure 2D) results clearly demonstrate separation between the healthy and infected groups in both positive and negative ion modes, indicating significant metabolic differences between the two groups.

### 3.3. Differential Metabolite Identification and Volcano Plot Analysis

**Figure 3:**
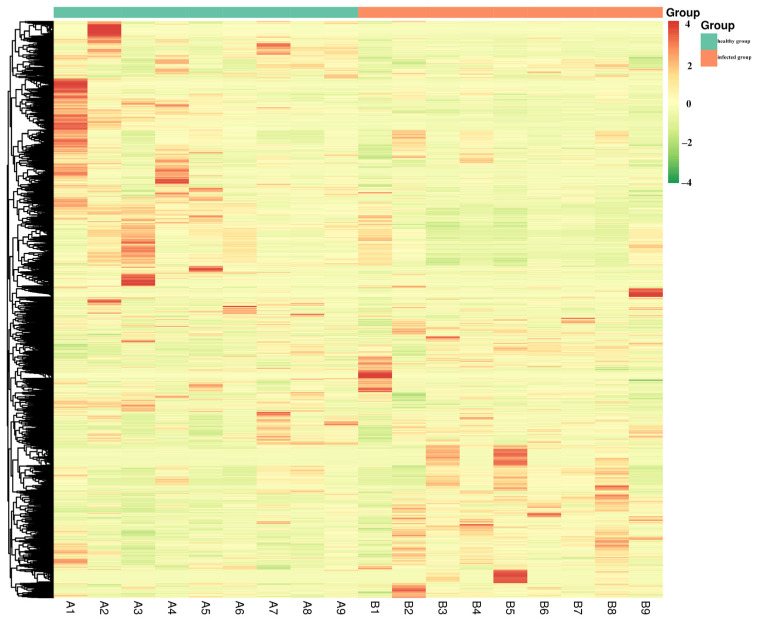
Heat map of DEM. The horizontal axis represents individual samples, labeled A1 to A9 (healthy group, blue-green bar) and B1 to B9 (infected group, red bar). The vertical axis represents differentially abundant metabolites identified by multivariate statistical analysis. “Group” refers to the sample grouping. Different colors represent the values obtained after normalization of relative content, with red indicating high content and green indicating low content. The selection criteria for differential metabolites were VIP > 1 and *p* < 0.05.

The hierarchical clustering heatmap (Figure 3) presents joint clustering of samples and detected metabolites. The left dendrogram groups metabolites based on similarity, while the top dendrogram clusters samples. Sample annotations indicate group identity: green for healthy and red for infected. The color intensity of each tile reflects the relative abundance, with deeper red indicating stronger upregulation and deeper green representing stronger downregulation. The distinct color patterns observed between the two groups highlight substantial, systematic differences in metabolite levels.

**Figure 4:**
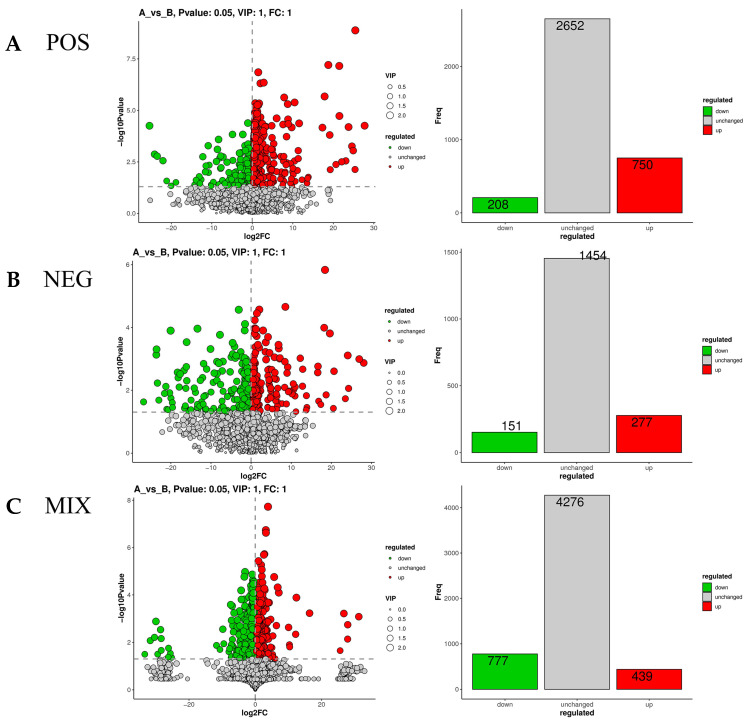
Volcano plot of metabolites. (**A**) Volcano plot of metabolites in positive-ion mode. (**B**) Volcano plot of metabolites in negative-ion mode. (**C**) Volcano plot of metabolites in mixed-ion mode. Significant DEM (VIP > 1 and *p* < 0.05) are in red (upregulated) and green (downregulated), whereas other metabolites are colored in gray.

Based on FDR correction (q < 0.05) and VIP > 1 criteria, a total of 1216 significantly different metabolites were identified in the mixed ion mode. Among these, 439 metabolites were significantly upregulated in the infected group, and 777 metabolites were significantly downregulated. A volcano plot (Figure 4) was generated for the selected differential metabolites, with each dot representing a metabolite. The five most significantly upregulated metabolites were Eremosulphoxinolide A, Atrovirinone, Pelargonidin 3-O-[b-D-Glucopyranosyl-(1->2)-[4-hydroxy-3-methoxy-(E)-cinnamoyl-(->6)]-b-D-glucopyranoside]5-O-b-D-glucopyranoside, PC(16:1(9Z)/22:2(13Z,16Z)), and Trihexosylceramide (d18:1/25:0). The five most significantly downregulated metabolites were Mifepristone, Isovalerylglutamic acid, PI(18:0/16:2(9Z,12Z)), Ganolucidic acid D, and Tragopogonsaponin Q.

### 3.4. Top Differential Metabolites and Their Correlation Patterns

**Figure 5:**
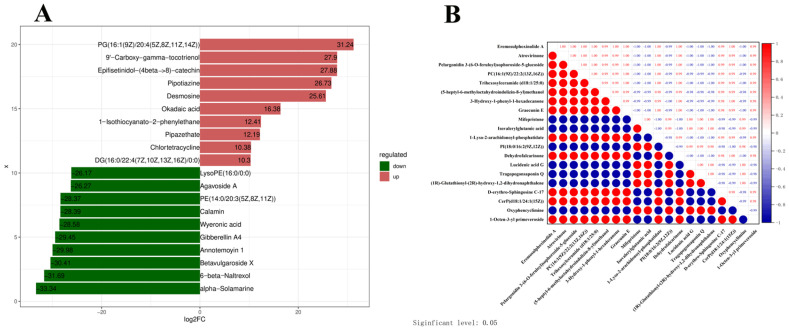
Analysis of metabolomic signatures and correlations of the 20 most significant differential metabolites (DEMs) in cattle during BPIV3 infection. (**A**) Twenty most significant DEM at day 10 during BPIV3 infection. The horizontal axis represents the log2FC of DEM, red represents an increase in metabolite content, and green represents a decrease in metabolite content. (**B**) the correlation among the top 20 significantly upregulated differential metabolites during BPIV3 infection. When two metabolites are positively correlated, their correlation coefficient approaches 1, indicating that their trends change in the same direction. When negatively correlated, the coefficient approaches −1, indicating that their trends change in opposite directions.

The fold change (FC) in metabolite quantification between the infected and healthy groups, expressed as log_2_FC, was calculated and compared. The top 10 upregulated and downregulated metabolites in the infected group compared to the healthy group are presented in Figure 5A, with red indicating upregulation (log_2_FC > 0) and green indicating downregulation (log_2_FC < 0). The upregulated metabolites include PG (16:1(9Z)/20:4(5Z, 8Z, 11Z, 14Z)), 9′-Carboxy-gamma-tocotrienol, Epifisetinidol-(beta->8)-catechin, Pipolizine, Desmosine, Okadaic acid, 1-Isothiocyanato-2-phenylethane, Pipazethate, Chlortetracycline, and DG (16:0; 22:4(7Z, 10Z, 13Z, 16Z)/0:0). The downregulated metabolites include alpha-Solamarine, 6-beta-Naltrexol, Betavulgaroside X, Anemtomycin 1, Gibberellin A4, Wyeronic acid, Calamin, PE (14:0/20:3(5Z, 8Z, 11Z)), Agavoside A, and LyoPE (16:0/0:0).

The top 20 most significantly different metabolites identified in the mixed-ion mode are listed in Table 2, with notable upregulation of metabolites such as Eremosulphoxinolide A, Atrovirinone, Pelargonidin 3-O-[β-D-Glucopyranosyl-(1->2)-[4-hydroxy-3-methoxy-(E)-cinnamoyl-(->6)]-β-D-glucopyranoside]5-O-β-D-glucopyranoside, PC(16:1(9Z)/22:2 (13Z,16Z)), Trihexosylceramide (d18:1/25:0), and (5-heptyl-6-methyloctahydroindolizin-8-yl)methanol, among others. In contrast, metabolites such as Mifepristone and Isovalerylglutamic acid are significantly downregulated. Of the top 20 differential metabolites, six are lipids, including PC(16:1(9Z)/22:2(13Z,16Z)), Trihexosylceramide (d18:1/25:0), 1-Lyso-2-arachidonoyl-phosphatidate, PI(18:0/16:2(9Z,12Z)), D-erythro-Sphingosine C-17, and CerP(d18:1/24:1(15Z)).

Metabolites may exhibit either synergistic or antagonistic relationships. When metabolites show similar trend changes (i.e., upregulated or downregulated together), they are positively correlated. Conversely, if their trends change in opposite directions, they are negatively correlated. Highly positively correlated metabolite clusters may indicate involvement in shared metabolic pathways or common regulatory mechanisms, while highly negatively correlated clusters may suggest antagonistic or competitive interactions. Figure 5B illustrates the correlation of the top 20 most significantly upregulated differential metabolites. In this figure, a correlation value approaching 1 indicates a strong positive correlation, while a value approaching −1 reflects a strong negative correlation.

Among the differential metabolites, lipids represent a significant category. Notably, PC(16:1(9Z)/22:1(13Z)) is highly positively correlated with Trihexosylceramide (d18:1/25:0), while 1-Lyso-2-arachidonoyl-phosphatidate, PI(18:0/16:2(9Z,12Z)), D-erythro-Sphingosine C-17, and Cer(d18:1/24:1(15Z)) exhibit strong negative correlations. These correlations suggest that, during BPIV3 infection, there are intricate and coordinated changes in these lipid metabolites, potentially linked to the infection’s progression.

### 3.5. KEGG Enrichment and Directionality Across Ion Modes Reveal Network-Level Metabolic Remodeling in BPIV3-Infected Simmental Cattle

**Figure 6:**
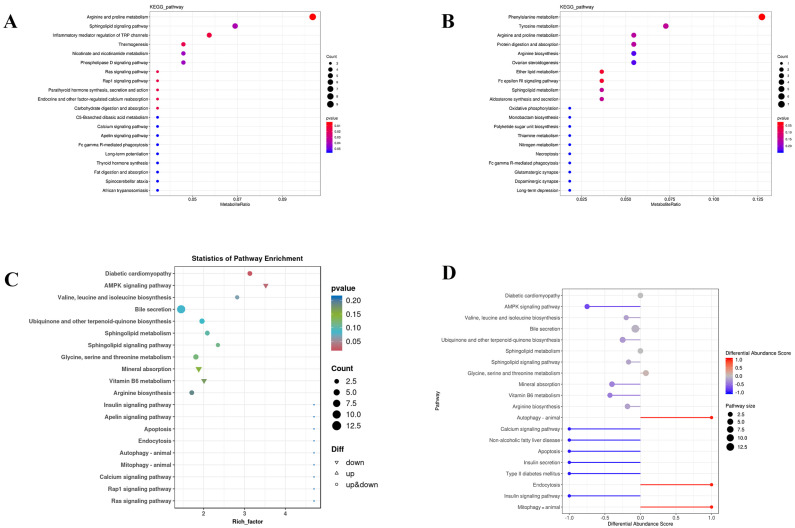
Enriched metabolic pathways. (**A**) Enriched metabolic pathways in positive-e-ion modes and (**B**) negative-ion modes; (**C**) The vertical axis displays the names of the differential pathways, ranked by p-value, while the horizontal axis shows the differential rich factor. This score indicates the overall trend of metabolic changes within each pathway; (**D**) Differential abundance scores of different metabolic pathways under mixed ion mode. The vertical axis displays the names of the differential pathways, ranked by *p*-value, while the horizontal axis shows the differential abundance score. This score indicates the overall trend of metabolic changes within each pathway. A score of 1 signifies an upregulation of all identified metabolites in the pathway, whereas a score of −1 indicates a downregulation of all identified metabolites.

KEGG metabolic pathway enrichment analysis was conducted on the differential metabolites identified in BPIV3-infected Simmental cattle compared to healthy controls. The analysis was based on the list of significantly different metabolites after FDR correction (q < 0.05), with statistical significance set at *p* < 0.05. Enriched pathways in both positive and negative ion modes are summarized in Figure 6. In the positive ion mode (Figure 6A), the most enriched pathways include parathyroid hormone synthesis, secretion, and action, phospholipase D signaling pathway, and nicotinate and nicotinamide metabolism. In the negative ion mode (Figure 6B), the most enriched pathways include long-term depression, dopaminergic synapse, and ovarian steroidogenesis, among others. Figure 6C shows the enrichment dot plot for the mixed ion mode, where the size of each dot is proportional to the scale of the metabolic pathway. The numbers on the x-axis represent the proportion of differential metabolites relative to the total metabolites within each pathway. The differential pathways between the healthy and infected groups are shown by the differential abundance scores (Figure 6D). The vertical axis lists the names of the top 20 differential metabolic pathways, arranged in descending order based on the differential abundance score. The horizontal axis represents the Differential Abundance Score (DAS), with the size of each dot reflecting the scale of the metabolic pathway. A DAS closer to 1 indicates a higher proportion of upregulated differential metabolites in the pathway, while a DAS closer to −1 indicates a higher proportion of downregulated metabolites. Among the pathways displayed, autophagy, apoptosis, and mitophagy have differential abundance scores approaching 1, suggesting an enrichment of upregulated metabolites in these pathways.

**Figure 7:**
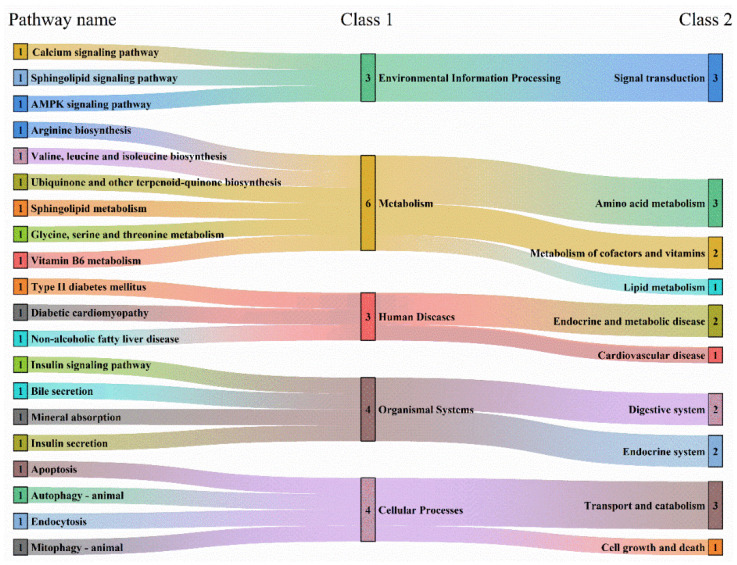
Sankey diagram. Pathways are listed on the left. The colored streams link each pathway to its primary KEGG class (Class 1, middle) and then to a more specific subclass (Class 2, right). The color of each stream represents its associated KEGG Class 1 category (e.g., blue-green for *Environmental Information Processing*, yellow-orange for *Metabolism*, and pink-red for *Human Diseases*), providing a visual grouping of the functional modules. The width of a stream corresponds to the number or strength of associations within that category.

The basic information for the 20 metabolic pathways is summarized in Table 3, while the relationships among the differential metabolic pathways are illustrated more intuitively in a Sankey diagram (Figure 7). Among the top 20 enriched pathways, six belong to the Metabolism category, including Arginine biosynthesis, Valine, leucine, and isoleucine biosynthesis, Ubiquinone and other terpenoid-quinone biosynthesis, Sphingolipid metabolism, Glycine, serine, and threonine metabolism, and Vitamin B6 metabolism.

### 3.6. Global Behavior of Phosphatidylinositol Species

To determine whether phosphatidylinositol (PI) depletion represents a global feature rather than a single-species effect, we performed targeted univariate analysis of all annotated PI species and related metabolites in the dataset. In total, 19 distinct PI species were identified in serum. Among these, 7 species showed significant decreases in BPIV3-infected animals compared with healthy controls, including PI(16:0/18:0), PI(16:0/18:1), PI(16:0/20:4), PI(18:0/16:2), PI(18:0/20:3), PI(18:2/20:1) and PI(22:3/18:3), with log2 fold changes ranging from −0.57 to −9.79 (P = 0.020 − 1.71 × 10^−5^). Nine PI species exhibited no significant change, and three species showed inconsistent trends between groups. Overall, the direction of change across the PI panel was biased toward down-regulation. In contrast, the PI-derived metabolite arachidonic acid did not change significantly (log2FC = 0.034, *p* = 0.70), and all four annotated lysophosphatidylinositol/phosphatidic acid (LPI/PA) intermediates also remained statistically unchanged (Table 3).

## 4. Discussion

This study, based on untargeted metabolomics, revealed a systematic remodeling of serum lipid profiles in Simmental cattle infected with BPIV3. PCR was employed to confirm the infection status (Figure 1). The distinction of metabolic profiles between the infected and healthy groups, along with model stability, was supported by correlation analysis, principal component analysis (PCA), orthogonal partial least squares discriminant analysis (OPLS-DA), permutation tests (Figure 2A–D), and clustering heatmaps (Figure 3), providing a reliable foundation for subsequent differential screening and mechanistic interpretation.

Phospholipids, as the primary building blocks of the lipid bilayer in cell mem-branes, play a critical role in maintaining membrane structural integrity, fluidity, and the accessibility of signaling molecules through their metabolic state [10,11,12,13]. In this study, PG(16: 1 (9Z)/20: 4(5Z, 8Z, 11Z, 14Z)) and PC(16:1(9Z)/22:2(13Z,16Z)) were significantly upregulated (log_2_FC = 2.76, *p* < 0.001), while phosphatidylinositol PI(18:0/16:2(9Z,12Z)) was markedly downregulated (log_2_FC = −3.29, *p* < 0.001) (Figure 4, Table 2), indicating that BPIV3 remodels the host membrane system by differentially regulating phospholipid subclasses.

The targeted analysis of PI species further refines the interpretation of our lipidomic findings. Although not all PI molecules were significantly altered, the fact that 7 of 19 annotated species were decreased, while the remainder were largely unchanged or showed no consistent direction, indicates that PI depletion is not restricted to a single outlier but reflects a broader tendency toward contraction of the circulating PI pool in BPIV3-infected cattle. By contrast, the absence of significant changes in arachidonic acid and LPI/PA intermediates suggests that classical PI breakdown products do not accumulate in serum, which may point to rapid downstream metabolism or to compartmentalized PI turnover at specific membrane sites that is only partially captured systemically. Taken together, these patterns are consistent with a selective reorganization of PI-centered lipid networks, superimposed on increased PG and PC species, and may be relevant to the modulation of membrane-associated signaling and innate antiviral responses during BPIV3 infection.

These phospholipid changes can be viewed in the broader context of viral “membrane hijacking.” Many positive-sense RNA viruses manipulate host phospholipid synthesis to generate viral replication complexes (VRCs) and vesicular transport structures [14,15,16]. For example, hepatitis C virus recruits phosphatidylinositol 4-kinase IIIα (PI4KIIIα) to enrich phosphatidylinositol 4-phosphate (PI4P) at endoplasmic reticulum membranes, promoting the formation of network-like structures that support viral RNA replication [17]. The envelope protein E of dengue virus (DENV) binds negatively charged phospholipids such as phosphatidylserine to facilitate fusion with host cell membranes [18,19], and DENV infection has been shown to induce substantial phospholipid reprogramming in both mosquito vectors and mammalian hosts [20,21,22,23,24]. Modulation of de novo phospholipid synthesis can influence viral yield [25], highlighting the functional relevance of phospholipid metabolism in positive-sense RNA virus replication. In this framework, the increased PG and PC species observed here may increase the availability of lipid substrates compatible with the formation of replication- or budding-related membrane microdomains, whereas decreased PI could, in principle, destabilize PI4P-enriched domains and thereby influence lipid raft organization and BPIV3 HN protein–mediated membrane fusion [3]. These mechanistic links remain hypothetical and require direct experimental validation.

Negative-sense RNA viruses also rely on host membranes, but the magnitude and pattern of lipid remodeling appear more restrained than in many positive-sense RNA virus models. Respiratory syncytial virus (RSV), influenza virus, and Ebola virus assemble progeny virions at the plasma membrane [10,11,12,13,14,15,16,17]. In influenza virus infection, host lipid metabolism is markedly perturbed [18,19], and particle assembly occurs at cholesterol- and sphingolipid-rich regions of the plasma membrane (budozones) that share key features with lipid rafts and support interactions between viral and host proteins [12,20,21]. In addition, acidic phospholipids are important determinants of influenza virus assembly and budding, and their abundance can be modulated during infection [22,23]. Newcastle disease virus (NDV) infection has been reported to deplete cellular lipids and activate genes related to the sphingolipid “rheostat,” with upregulation of sphingomyelin synthase promoting ceramide accumulation and supporting viral assembly and replication [24]. Experimental BPIV3 inoculation in calves was previously shown to decrease the proportion of phosphatidylcholine in lung lavage fluid [25], further indicating that negative-sense RNA viruses can selectively reshape surfactant and membrane-associated lipid pools, even though the overall extent of lipidome remodeling is often less dramatic than that described for many positive-sense RNA viruses. Importantly, our data are derived from serum lipids rather than direct membrane extracts, and therefore should be interpreted as reflecting systemic lipid reprogramming associated with BPIV3 infection rather than direct proof of extensive cellular membrane remodeling.

In vivo lipidomic studies further indicate that systemic phospholipid remodeling is a recurrent feature of RNA virus infection. In chickens, Dai et al. combined targeted metabolomics with single-cell transcriptomics and showed that Newcastle disease virus (NDV) infection was accompanied by broad changes in plasma phospholipid metabolism, with the velogenic Herts/33 strain associated with global decreases in ceramide, diacylglycerol, triglycerides, sphingomyelin, phosphatidylcholine (PC), phosphatidylethanolamine (PE), phosphatidylinositol (PI), phosphatidylserine (PS) and phosphatidic acid (PA), whereas the lentogenic LaSota strain tended to induce opposite trends; moreover, plasma diacylglycerol levels correlated with the expression of metabolic enzymes in pulmonary endothelial and interstitial cells, linking circulating lipids to tissue-level metabolic reprogramming [24]. In adult patients with laboratory-confirmed influenza A(H1N1), Li et al. reported pronounced disturbances of plasma glycerophospholipid metabolism, in which more than 80% of differential glycerophospholipids—including several PC and LysoPC species—were down-regulated relative to healthy controls and showed graded changes across severity strata, with LysoPC(20:0/0:0) negatively associated with inflammatory markers and positively associated with lymphocyte counts [26]. Together with earlier work highlighting the contribution of phospholipid and sphingolipid remodeling to influenza pathogenesis and host responses [19,27,28,29], these findings suggest that the alterations in circulating PC, PI and related subclasses observed in BPIV3-infected cattle are part of a broader pattern of virus-associated perturbations in host lipid homeostasis, and may reflect a combination of local membrane remodeling in target tissues and systemic inflammatory or immune status rather than a BPIV3-specific phenomenon.

Phosphatidylinositol itself has been identified as an anionic pulmonary surfactant lipid with strong antiviral activity against RSV. Numata et al. demonstrated that PI liposomes bind RSV with high affinity, reduce IL-8 production, and decrease RSV infection by more than 10^3^-fold in BEAS-2B cells; in mice, co-administration or prophylactic administration of PI markedly lowered viral load, inflammatory cell recruitment and tissue damage [30]. Together with phosphatidylglycerol, PI thus functions as a component of innate immune defense in the lung. In this light, the depletion of multiple PI species and the simultaneous activation of the sphingolipid axis observed in our study may indicate that BPIV3 infection engages lipid pathways that are also implicated in other negative-sense RNA virus infections and in surfactant-mediated antiviral protection. However, further work using cell-based infection models and compartment-specific lipidomics will be necessary to clarify how serum PI changes relate to local lipid dynamics at the respiratory epithelium and immune effector sites.

Beyond glycerophospholipids, we also observed a characteristic pattern within the sphingolipid axis. The increase in trihexosylceramide, sphingosine and several ceramide species, together with changes in sphingomyelin, is compatible with activation of a ceramide-centered “rheostat” similar to that described for NDV [24]. Ceramides and complex glycosphingolipids are known to participate in raft organization, receptor clustering, and apoptotic or stress signaling, and have been implicated in the life cycles of multiple respiratory viruses. In our data, the relative enrichment of ceramide and sphingosine, accompanied by altered sphingomyelin and trihexosylceramide, may reflect a shift toward a pro-fusion and pro-assembly lipid environment, as well as modulation of inflammatory signaling in BPIV3-infected cattle. From a translational perspective, these observations raise the possibility that targeting sphingolipid synthesis (for example, by inhibiting specific sphingomyelin synthase isoforms) or ceramide trafficking could modulate BPIV3 assembly and budding, although this notion remains to be tested experimentally.

In addition to lipid changes, the downregulation of branched-chain amino acid (BCAA)-related metabolites, such as isovaleric acid and 3-methyl-2-oxovaleric acid, suggests that BPIV3 infection is associated with altered amino acid and energy metabolism. BCAAs are important substrates for the tricarboxylic acid (TCA) cycle and can influence cellular redox balance, mTOR/AMPK signaling, and autophagy. The decreased levels of BCAA catabolites in infected animals may indicate increased utilization of these substrates for energy production or stress adaptation, or a shift in hepatic and muscular amino acid handling during infection. Previous studies have shown that activation of AMPK and autophagy can either support or restrict viral replication depending on the virus and cellular context. In BPIV3 infection, it will be important to determine whether changes in BCAA metabolism are linked to AMPK signaling and autophagic flux in respiratory epithelial cells and immune cells, and whether these pathways influence viral replication efficiency. Our metabolomic data provide a rationale for such mechanistic studies but do not yet allow for definitive conclusions about causal relationships.

We acknowledge several limitations of this study. First, serum lipids mainly reflect lipoprotein-associated pools and systemic metabolic responses rather than direct extracts from specific cellular membranes. As a result, the observed alterations in PC, PG, PI and sphingolipids should be interpreted as evidence of systemic lipid reprogramming associated with BPIV3 infection, not as direct proof of localized membrane remodeling. Second, because the animals included in this study were naturally infected and exhibited clinical signs, it is difficult to fully disentangle the effects of viral replication from those of reduced feed intake, fever, inflammation and other stressors that can independently influence host metabolism, particularly lipid and energy pathways. Finally, our analyses were performed at a single time point, which precludes assessment of the temporal dynamics of metabolic changes during the course of infection and recovery.

While we cannot completely separate the contributions of BPIV3 infection and illness-related metabolic stress, the resemblance of our lipidomic findings to patterns reported in other viral infections is compatible with a role for direct viral reprogramming of host lipid metabolism, in addition to generalized responses to disease. In conclusion, despite these limitations, our study provides an initial system-level view of serum lipid and metabolite perturbations in BPIV3-infected cattle, highlighting coordinated alterations in PI, PG, PC and sphingolipid pathways, as well as changes in BCAA-related metabolites. These results generate testable hypotheses regarding the involvement of specific lipid and amino acid networks in BPIV3 pathogenesis and may help guide future work aimed at identifying biomarkers of infection and potential metabolic intervention targets.

## Figures and Tables

**Table 1:** Detection primers used in this study.

Primers	Sequence	Target Gene	Product Size
BVDV-F	ATGCCCWTAGTAGGACTAGCA	5′UTR	288 bp
BVDV-R	TCAACTCCATGTGCCATGTAC		
BRSV-F	GGCAACAGAGTTATTCCA	F	418 bp
BRSV-R	TTTGTCTTCCCATAGCAT		
BoHV1-F	CGACGAGGAGACGCAGTTGG	gE	225 bp
BoHV1-R	CGCACCGATAGCAGAAAGGAAT		
BPIV3-F	CCGGGGATTTATTATAAAGGT	HN	122 bp
BPIV3-R	CTCTCTGTGTTTTGCCTG		

**Table 2:** The top 20 most significantly differential metabolites based on *p*-value in mixed-ion mode.

Name	log2FC	*p* Value	VIP	Regulate
Eremosulphoxinolide A	3.838740389	1.87 × 10^−8^	2.438413625	up
Atrovirinone	3.186871059	1.82 × 10^−7^	2.3819343	up
Pelargonidin 3-O-[b-D-Glucopyranosyl-(1->2)-[4-hydroxy-3-methoxy-(E)-cinnamoyl-(->6)]-b-D-glucopyranoside] 5-O-b-D-glucopyranoside	3.216980466	2.37 × 10^−7^	2.369863889	up
PC(16:1(9Z)/22:2(13Z,16Z))	2.760474591	1.89 × 10^−6^	2.246792773	up
Trihexosylceramide (d18:1/25:0)	2.591260164	2.01 × 10^−6^	2.290914319	up
(5-heptyl-6-methyloctahydroindolizin-8-yl)methanol	0.962948371	3.72 × 10^−6^	2.24294215	up
3-Hydroxy-1-phenyl-1-hexadecanone	1.772556758	5.44 × 10^−6^	2.260481151	up
Graecunin E	1.962276436	8.58 × 10^−6^	2.241946324	up
Mifepristone	−3.085137439	1.07 × 10^−5^	2.252322884	down
Isovalerylglutamic acid	−0.922129751	1.36 × 10^−5^	2.178265457	down
1-Lyso-2-arachidonoyl-phosphatidate	1.991576223	1.64 × 10^−5^	2.197673812	up
PI(18:0/16:2(9Z,12Z))	−3.286098014	1.71 × 10^−5^	2.274602619	down
Dehydrofalcarinone	5.696407062	1.76 × 10^−5^	2.37277912	up
Lucidenic acid G	−0.714955069	2.40 × 10^−5^	2.185338404	down
Tragopogonsaponin Q	−0.470967794	2.80 × 10^−5^	2.16494858	down
(1R)-Glutathionyl-(2R)-hydroxy-1,2-dihydronaphthalene	−0.714153379	2.98 × 10^−5^	2.162099199	down
D-erythro-Sphingosine C-17	2.180928816	3.03 × 10^−5^	2.194792841	up
CerP(d18:1/24:1(15Z))	1.099136311	3.09 × 10^−5^	2.210163025	up
Oxyphencyclimine	−0.592909062	3.46 × 10^−5^	2.123981337	down
1-Octen-3-yl primeveroside	1.298369398	3.58 × 10^−5^	2.147241875	up

**Table 3:** Enrichment analysis of KEGG Pathway.

Pathway Name	KEGG ID	Class 1	Class 2
Diabetic cardiomyopathy	map05415	Human Diseases	Cardiovascular disease
AMPK signaling pathway	map04152	Environmental Information Processing	Signal transduction
Valine, leucine and isoleucine biosynthesis	map00290	Metabolism	Amino acid metabolism
Bile secretion	map04976	Organismal Systems	Digestive system
Ubiquinone and other terpenoid-quinone biosynthesis	map00130	Metabolism	Metabolism of cofactors and vitamins
Sphingolipid metabolism	map00600	Metabolism	Lipid metabolism
Sphingolipid signaling pathway	map04071	Environmental Information Processing	Signal transduction
Glycine, serine and threonine metabolism	map00260	Metabolism	Amino acid metabolism
Mineral absorption	map04978	Organismal Systems	Digestive system
Vitamin B6 metabolism	map00750	Metabolism	Metabolism of cofactors and vitamins
Arginine biosynthesis	map00220	Metabolism	Amino acid metabolism
Autophagy–animal	map04140	Cellular Processes	Transport and catabolism
Calcium signaling pathway	map04020	Environmental Information Processing	Signal transduction
Non-alcoholic fatty liver disease	map04932	Human Diseases	Endocrine and metabolic disease
Apoptosis	map04210	Cellular Processes	Cell growth and death
Insulin secretion	map04911	Organismal Systems	Endocrine system
Type II diabetes mellitus	map04930	Human Diseases	Endocrine and metabolic disease
Endocytosis	map04144	Cellular Processes	Transport and catabolism
Insulin signaling pathway	map04910	Organismal Systems	Endocrine system
Mitophagy–animal	map04137	Cellular Processes	Transport and catabolism

## Data Availability

The original contributions presented in this study are included in the article. Further inquiries can be directed to the corresponding author.

## Abbreviations

The following abbreviations are used in this manuscript:

BPIV3	Bovine Parainfluenza Virus Type 3
BRDC	Bovine Respiratory Disease Complex
UPLC-QTOF-MS/MS	ultra-performance liquid chromatography-quadrupole time-of-flight mass spectrometry
PC	phosphatidylcholine
PG	phosphatidylglycerol
PI	phosphatidylinositol
HN	hemagglutinin-neuraminidase
ER	endoplasmic reticulum
PCR	polymerase chain reaction
IBRV	infectious bovine rhinotracheitis virus
BVDV	bovine viral diarrhea virus
BRSV	bovine respiratory syncytial virus
OPLS-DA	orthogonal partial least squares discriminant analysis
PCA	principal component analysis
VIP	variable importance in projection
KEGG	Kyoto Encyclopedia of Genes and Genomes
FC	fold change
FDR	false discovery rate
DEM	differential metabolites
LPA	lysophosphatidic acid
PRRSV	porcine reproductive and respiratory syndrome virus
RIG-I	retinoic acid-inducible gene I
SMS2	sphingomyelin synthase 2
BCAA	branched-chain amino acids
AMPK	AMP-activated protein kinase
